# An Efficient Broadband Adaptive Beamformer without Presteering Delays

**DOI:** 10.3390/s21041100

**Published:** 2021-02-05

**Authors:** Ming Zhang, Xiaojian Wang, Anxue Zhang

**Affiliations:** School of Information and Communications Engineering, Xi’an Jiaotong University, Xi’an 710049, China; wangxiaojian@stu.xjtu.edu.cn (X.W.); anxuezhang@xjtu.edu.cn (A.Z.)

**Keywords:** sensor array, presteering delays, time domain constraint, NLMS adaptive algorithm

## Abstract

Broadband adaptive beamformers have been widely used in many areas due to their ability of filtering signals in space domain as well as in frequency domain. However, the space-time array employed in broadband beamformers requires presteering delays to align the signals coming from a specific direction. Because the presteering delays are direction dependent, it is difficult to make precise delays in practice. A common way to eliminate the presteering delays is imposing constraints on the weight vector of the space-time array. However, the structure of the constraint matrix is not taken into account in the existing methods, leading to a computational complexity of $O(N^{2})$ when updating the weight vector. In this paper, we describe a new kind of constraint method in time domain that preserves the block diagonal structure of the constraint matrix. Based on this structure, we design an efficient weight vector update algorithm that has a computational complexity of O(N). In addition, the proposed algorithm does not contain matrix operations (only scalar and vector operations are involved), making it easy to be implemented in chips such as FPGA. Moreover, the constraint accuracy of the proposed method is as high as the frequency constraint method when the fractional bandwidth of the signal is smaller than 10%. Numerical experiments show that our method achieves the same performance of the state-of-the-art methods while keeping a simpler algorithm structure and a lower computational cost.

## 1. Introduction

Beamforming has become a fundamental technique for sensor arrays and received considerable attention in recent decades [1,2,3,4,5]. Besides providing the ability of performing spatial filtering, the broadband beamformers can also control the frequency response in a given direction [6], which makes it a power tool in various areas such as radar, sonar, mobile communications, satellite navigations, and radio astronomy. The space-time adaptive processing is one of the widely used approaches to implement broadband beamforming. An adaptive broadband beamformer based on space-time array was proposed in [7], which uses the least mean square (LMS) algorithm to compute the weight vector of the array. This method has been used in a wide range of applications due to its low computational complexity and good numerical stability. Another commonly used technique for broadband beamforming is the space-frequency adaptive processing [8,9], which splits the frequency band of the incident signal into several narrow subbands and then uses narrowband beamformers to deal with the narrowband signals. The space-frequency processing has a faster convergence rate than space-time processing when the number of taps is large (e.g., hundreds or thousands). Nevertheless, space-frequency beamformer has a larger processing delay than space-time beamformer [10,11]. Therefore, space-time adaptive processing is still the first choice for delay sensitive applications such as satellite navigations [12,13].

To obtain a desired frequency response in the direction of the signal of interest (SOI), constraints should be imposed on the weight vector of the beamformer. Most of these constraints require that the array has been presteered toward the direction of SOI. However, it is difficult to implement precise delays in practice [14,15], especially for the situations that sensor array has to form multiple beams [16,17]. In addition, the presteering errors may cause signal cancellation in the direction of SOI, leading to performance degradation of the beamformer [18,19]. Therefore, it is very important to eliminate the presteering delays in broadband beamformers. A type of convolution constraint method was proposed in [20] to remove the presteering delays. This method has a simple procedure to implement; however, it is computationally expensive. The authors in [21] described another technique to remove the presteering delays, which multiplies the received signal by a matrix whose elements are the inverse Fourier transform of the steering vector. Thus, it is computationally expensive too. By introducing a set of frequency domain constraints (FDC), the authors in [22] developed a simple technique to eliminate the presteering delays, which requires less tapped delay-lines (TDLs) than the convolution constraint method. Moreover, it can incorporate the sensor patterns into the beamforming algorithm and thus can be applied to conformal arrays [23]. The FDC method based on generalized sidelobe canceller (FDC-GSC) was presented in [24], which is mathematically equivalent to FDC but with fewer computations. To further reduce the number of TDLs, infinite impulse response filter can be used [25,26]. However, all of these methods destroy the block diagonal structure of the constraint matrix, making the computational complexity of weight vector update increased from O(N) to $O(N^{2})$.

To reduce the computational complexity of weight vector update, we design a new kind of time domain approximate constraint (TDAC) method, which does not require the presteering delays while preserving the block diagonal structure of the constraint matrix. The constraint accuracy of TDAC is as high as the FDC method if the fractional bandwidth is smaller than 10%. This is a reasonable assumption because the fractional bandwidth of most radar and communication systems is smaller than 10%. Moreover, by exploiting the block diagonal structure of the constraint matrix, a new efficient weight vector update algorithm with a complexity of O(N) is also developed. In fact, the computations involved in each iteration of the proposed algorithm is only half of that of the conventional Frost algorithm (CFA). Both LMS and normalized LMS (NLMS) algorithms can be used to update the weight vector of the space-time array. We shall study the NLMS-based algorithm in this paper because the one based on LMS can be easily obtained from NLMS.

The rest of this paper is organized as follows. Section 2 reviews the signal model of space-time array and the conventional Frost algorithm. Section 3 describes the details of the proposed method, including algorithm design, geometrical interpretation, and complexity analysis. Numerical simulations are presented in Section 4, followed by conclusions in Section 5.

Notation: We use lowercase letters (*a*), lowercase boldface letters (a), uppercase boldface letters (A), and uppercase calligraphy letters (A) to represent scalars, vectors, matrices and sets, respectively. The superscripts ${\left(\right)}^{*}$, ${\left(\right)}^{T}$, ${\left(\right)}^{H}$, ${\left(\right)}^{⊥}$ and ${\left(\right)}^{-1}$ stand for complex conjugate, transpose, conjugate transpose, orthogonal complement, and inverse, respectively. The symbol ⊗ denotes the Kronecker product, ∥·∥ represents the Euclid norm, E(·) stands for the mathematical expectation, and O(·) means “on the order of”.

## 2. Space-Time Adaptive Beamformer

This section reviews the signal model of space-time array and the conventional Frost algorithm, including space-time steering vector, frequency-wavenumber response (FWR), and weight vector update methods of Frost algorithm based on LMS and NLMS.

### 2.1. Signal Model of Space-Time Array

**Figure 1:**
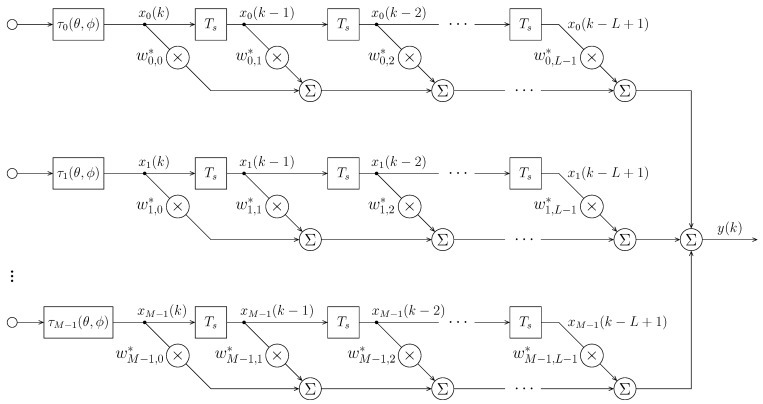
The structure of a space-time array (see also [7]).

Figure 1 shows a space-time array with *M* sensors and each sensor is followed by a time delay unit and a finite impulse response (FIR) filter of *L* taps (the radio frequency chain and the analog-to-digital converter are omitted for simplicity). The aim of presteering delays $\tau_{m}\left(\theta ,\phi \right)$ is to align the signal coming from the direction of SOI so that there is no phase difference among the SOI received by different sensors. The total degrees of freedom of the array, i.e., the number of free weights [6], is N=ML, and the N×1 baseband signal vector at the *k*th time instant is given by

(1) $$x\left(k\right)={\left[x_{0}^{T}\left(k\right),\;x_{1}^{T}\left(k\right),\;\cdots ,\;x_{L-1}^{T}\left(k\right)\right]}^{T}\;,$$

where $x_{l}\left(k\right)={\left[x_{0}\left(k-l\right),\cdots ,x_{M-1}\left(k-l\right)\right]}^{T}$ is the M×1 signal vector at the *l*th tap for l=0,1,⋯,L−1. Similarly, the N×1 weight vector is given by

(2) $$w={\left[w_{0}^{T},\;w_{1}^{T},\;\cdots ,\;w_{L-1}^{T}\right]}^{T}\;,$$

where $w_{l}={\left[w_{0,l},\cdots ,w_{M-1,l}\right]}^{T}$ is the M×1 weight vector at the *l*th tap.

If there are no presteering delays, the FWR of the array for the signal coming from (θ,ϕ) at frequency $\omega_{c}+\omega$ can be expressed as [27]

(3) $$\Gamma \left(\omega ,k\right)=\sum_{l=0}^{L-1}\sum_{m=0}^{M-1}w_{m,l}^{*}e^{-jk^{T}p_{m}}e^{-j\omega lT_{s}}=w^{H}v\left(\omega ,k\right)\;,$$

where $k=[\left(\omega_{c}+\omega \right)/c]a$ is the wavevector of the impinging signal, $\omega_{c}$ is the carrier frequency, ω is the baseband frequency, *c* is the speed of light, $a=-{\left[\sin\left(\theta \right)\cos\left(\phi \right),\sin\left(\theta \right)\sin\left(\phi \right),\cos\left(\theta \right)\right]}^{T}$ is the propagation direction, $p_{m}$ is the position vector of the *m*th sensor, and $T_{s}$ is the sampling period of the baseband signal. The steering vector v(ω,k) of the space-time array can be expressed as [28]

(4) $$v\left(\omega ,k\right)=v_{t}\left(\omega \right)⊗v_{s}\left(k\right)\;,$$

where

(5) $$v_{t}\left(\omega \right)={\left[1,\;e^{-j\omega T_{s}},\;\cdots ,\;e^{-j\left(L-1\right)\omega T_{s}}\right]}^{T}$$

is the temporal steering vector and

(6) $$v_{s}\left(k\right)={\left[e^{-jk^{T}p_{0}},\;e^{-jk^{T}p_{1}},\;\cdots ,\;e^{-jk^{T}p_{M-1}}\right]}^{T}$$

is the spatial steering vector.

### 2.2. The Conventional Frost Algorithm

The linearly constrained minimum variance beamformer is a widely used technique to suppress the interferences while keeping the SOI, which minimizes the output power of the array subject to *J* linear constraints as follows

(7) $$w=\arg\min w^{H}R_{x}w\;,\;s.t.\;C^{H}w=g\;,$$

where $R_{x}=E\left[x\left(k\right)x^{H}\left(k\right)\right]$ is the N×N space-time covariance matrix of the received signal, C is the N×J constraint matrix whose columns are linearly independent and g is the J×1 gain vector. The solution of (7) is given by [27]

(8) $$w_{o}=R_{x}^{-1}C{\left(C^{H}R_{x}^{-1}C\right)}^{-1}g\;.$$

It is not advisable to compute the weight vector by solving the above equation for real-time processing applications, because one has to estimate the covariance matrix $R_{x}$ and compute the matrix inversion (or solve linear systems of equations). Frost developed an adaptive approach to compute the weight vector based on the LMS algorithm as follows [7]:

(9) $$w\left(k+1\right)=w_{q}+P\left[w\left(k\right)-\mu y^{*}\left(k\right)x\left(k\right)\right]\;,$$

where μ is the step size parameter,

(10) $$w_{q}=C{\left(C^{H}C\right)}^{-1}g$$

is the quiescent weight vector in the column space of C, denoted by R(C), and

(11) $$P=I-C{\left(C^{H}C\right)}^{-1}C^{H}$$

is the projection matrix onto the orthogonal complement of R(C), denoted by $R^{⊥}\left(C\right)$. The normalized version of (9) is given by [29]

(12) $$w\left(k+1\right)=w_{q}+P\left[w\left(k\right)-\frac{\mu y^{*}\left(k\right)}{x^{H}\left(k\right)Px\left(k\right)}x\left(k\right)\right]\;.$$

## 3. The Proposed Method

Although the CFA (9) (or (12)) is simple, it requires the presteering delays. In this section, we first introduce a new kind of constraint for the space-time array, which eliminates the presteering delays and enables the weight vector to be updated efficiently. Then, inspired by [7], we give a geometric interpretation of the proposed algorithm. Finally, the comparison of computational complexity of our method and the existing methods is provided.

### 3.1. The Approximate Constraints in Time Domain

Suppose that the number of constraints *J* in (7) is equal to the number of taps *L* of the array, and let $w_{l}^{H}v_{s}\left(k\right)=g_{l}^{*}$, then the FWR (3) of the space-time array can be expressed as

(13) $$\Gamma \left(\omega ,k\right)=\sum_{l=0}^{L-1}w_{l}^{H}v_{s}\left(k\right)e^{-j\omega lT_{s}}=\sum_{l=0}^{L-1}g_{l}^{*}e^{-j\Omega l}\;,$$

where $\Omega =\omega T_{s}\in \left[-\pi ,\pi \right]$ is the normalized frequency [30]. To keep the symbols consistent with (7), we define $w_{l}^{H}v_{s}\left(k\right)=g_{l}^{*}$, i.e., $v_{s}^{H}\left(k\right)w_{l}=g_{l}$. If $g_{l}$ is independent of Ω, then Γ(ω,k) is equal to the discrete Fourier transform (DFT) of an FIR filter whose *l*th coefficient is equal to the inner product of $w_{l}$ and $v_{s}\left(k\right)$.

Equation (13) shows that the broadband beamformers can perform frequency filtering as well as spatial filtering. If the frequency response in the direction of SOI is determined by an FIR filter with coefficients $g={\left[g_{0},\cdots ,g_{L-1}\right]}^{T}$, we can impose constraints on $w_{l}$ in the form of $v_{s}^{H}\left(k\right)w_{l}=g_{l}$. However, because the wavevector k depends on baseband frequency ω, the coefficients $g_{l}$ in (13) depend on ω too. Nevertheless, for systems that operate with a small fractional bandwidth, the difference between k and $k_{0}=\left(\omega_{c}/c\right)a$ is small and k can be approximated by $k_{0}$. Thus, we obtain the following approximate constraints for the weight vector

(14) $$w_{l}^{H}v_{s}\left(k_{0}\right)=g_{l}^{*},\;l=0,1,\cdots ,L-1\;.$$

The above approximation is reasonable for many practical broadband systems that operate with a fractional bandwidth smaller than 10%, such as radar [28], satellite navigations [12], and wireless communications [31]. The benefit of this approximation is that it allows an efficient implementation of the weight vector update.

The constraints given by (14) can be rewritten as $C^{H}w=g$, which takes the same form of the constraint given in (7). The constraint matrix C has a block diagonal structure as follows

(15) $$C=\left[\begin{array}{cccc} v_{s}\left(k_{0}\right) & 0 & \cdots & 0\\ 0 & v_{s}\left(k_{0}\right) & \cdots & 0\\ \vdots & \vdots & \ddots & \vdots \\ 0 & 0 & \cdots & v_{s}\left(k_{0}\right) \end{array}\right]=I_{L}⊗v_{s}\left(k_{0}\right)\;,$$

where $I_{L}$ is the L×L identity matrix. The main computations of (12) come from the projection operation. Thus, we should derive a simple form for the projection matrix P. By using the properties ${\left(A⊗B\right)}^{H}=A^{H}⊗B^{H}$ and (A⊗B)(C⊗D)=(AC)⊗(BD) [32], we have

(16) $$w_{q}=C{\left(C^{H}C\right)}^{-1}g=\frac{1}{M}g⊗v_{s}\left(k_{0}\right)$$

and

(17) $$C{\left(C^{H}C\right)}^{-1}C^{H}=\frac{1}{M}I_{L}⊗\left[v_{s}\left(k_{0}\right)v_{s}^{H}\left(k_{0}\right)\right]\;.$$

Let

(18) $$Q=I_{M}-\frac{1}{M}v_{s}\left(k_{0}\right)v_{s}^{H}\left(k_{0}\right)=I_{M}-s\left(k_{0}\right)s^{H}\left(k_{0}\right)\;,$$

where $s\left(k_{0}\right)=v_{s}\left(k_{0}\right)/\sqrt{M}$ is the normalized spatial steering vector. Then the projection matrix P can be written as

(19) $$P=I_{N}-C{\left(C^{H}C\right)}^{-1}C^{H}=I_{L}⊗I_{M}-I_{L}⊗\left[s\left(k_{0}\right)s^{H}\left(k_{0}\right)\right]=I_{L}⊗Q\;.$$

Expressing the N×N projection matrix P by an M×M projection matrix Q is the key point of the efficient algorithm, which will be described in the next subsection.

### 3.2. An Efficient Weight Vector Update Algorithm

Because $w_{q}$ is independent of time instant *k*, only the second term on the right hand side of (12) needs updating. Let

(20) $$w_{a}\left(k+1\right)=P\left[w\left(k\right)-\frac{\mu y^{*}\left(k\right)}{p(k)}x\left(k\right)\right]=P\tilde{w}\left(k\right)\;,$$

where

(21) $$p\left(k\right)=x^{H}\left(k\right)Px\left(k\right)\;\mathrm{and}\;\tilde{w}\left(k\right)=w\left(k\right)-\frac{\mu y^{*}\left(k\right)}{p(k)}x\left(k\right)\;.$$

Then w(k+1) can be decomposed into two terms as follows

(22) $$w\left(k+1\right)=w_{q}+w_{a}\left(k+1\right)\;.$$

We call $w_{a}\left(k+1\right)$ the adaptive weight vector because it is updated adaptively according to the input signal vectors x(k).

Since P is the projection matrix onto $R^{⊥}\left(C\right)$ and $w_{q}\in R\left(C\right)$, we have ${\mathrm{Pw}}_{q}=0$. Thus,

(23) $$\begin{array}{cc} w_{a}\left(k+1\right) & =Pw\left(k\right)-[\mu y^{*}\left(k\right)/p\left(k\right)]Px\left(k\right)\\ \; & =P\left[w_{q}+w_{a}\left(k\right)\right]-\left[\mu y^{*}\left(k\right)/p\left(k\right)\right]\mathrm{Px}\left(k\right)\\ \; & =Pw_{a}\left(k\right)-\left[\mu y^{*}\left(k\right)/p\left(k\right)\right]\mathrm{Px}\left(k\right)\;. \end{array}$$

By using the idempotent property $P^{2}=P$ of the projection matrix [33], we have

(24) $$Pw_{a}\left(k\right)=P\left[P\tilde{w}\left(k-1\right)\right]=P\tilde{w}\left(k-1\right)=w_{a}\left(k\right)\;.$$

Hence,

(25) $$w_{a}\left(k+1\right)=w_{a}\left(k\right)-\frac{\mu y^{*}\left(k\right)}{p(k)}\mathrm{Px}\left(k\right)\;.$$

Next we show how to compute z(k)=Px(k) and $p\left(k\right)=x^{H}\left(k\right)\mathrm{Px}\left(k\right)$ efficiently. Because, as shown in Figure 1, $x_{l}\left(k\right)$ has a time delay structure $x_{l}\left(k\right)=x_{0}\left(k-l\right)$,

(26) $$z_{l}\left(k\right)≜{\mathrm{Qx}}_{l}\left(k\right)={\mathrm{Qx}}_{0}\left(k-l\right)=z_{0}\left(k-l\right)$$

has a similar time delay structure. It follows from (1) and (19) that

(27) $$\begin{array}{cc} z(k) & =\mathrm{Px}\left(k\right)={\left[z_{0}^{T}\left(k\right),\;z_{1}^{T}\left(k\right),\;\cdots ,\;z_{L-1}^{T}\left(k\right)\right]}^{T}\\ \; & ={\left[z_{0}^{T}\left(k\right),\;z_{0}^{T}\left(k-1\right),\;\cdots ,\;z_{0}^{T}\left(k-L+1\right)\right]}^{T}\;, \end{array}$$

where

(28) $$z_{0}\left(k\right)={\mathrm{Qx}}_{0}\left(k\right)=x_{0}\left(k\right)-s\left(k_{0}\right)\left[s^{H}\left(k_{0}\right)x_{0}\left(k\right)\right]\;.$$

Therefore, only $z_{0}\left(k\right)$ needs to be computed at each iteration, which involves 2M−1 complex additions and 2M complex multiplications. Although p(k) can be computed by $x^{H}\left(k\right)z\left(k\right)$ with N−1 complex additions and *N* complex multiplications. However, there exists a more efficient method to compute p(k) as follows

(29) $$p\left(k\right)=x^{H}\left(k\right)Px\left(k\right)=\sum_{l=0}^{L-1}x_{l}^{H}\left(k\right)z_{l}\left(k\right)=p\left(k-1\right)+x_{0}^{H}\left(k\right)z_{0}\left(k\right)-x_{L}^{H}\left(k\right)z_{L}\left(k\right)\;,$$

where the time delay properties $x_{l-1}\left(k-1\right)=x_{l}\left(k\right)$ and $z_{l-1}\left(k-1\right)=z_{l}\left(k\right)$ are used. Since $z_{0}\left(k\right)$ has already been calculated and each term on the right hand side of the above equation is real, there are only *M* complex additions and *M* complex multiplications in computing p(k) if we store the quantities $x_{0}^{H}\left(k\right)z_{0}\left(k\right)$.

**Figure 2:**
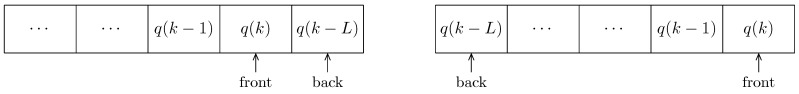
The circular array that stores $q\left(i\right)=x_{0}^{H}\left(i\right)z_{0}\left(i\right)$ for i=k, k−1, ⋯, k−L.

A circular array [34] is employed in our method to store the latest L+1 quantities of $x_{0}^{H}\left(i\right)z_{0}\left(i\right)$ for i=k, k−1, ⋯, k−L. The circular array, denoted by q, is shown in Figure 2, where the position *front* points at the current quantity q(k) and the position *back* points at the *L*th previous quantity q(k−L). When new data q(k) arrives, the circular array overwrites q(k−L) with q(k).

The final description of our algorithm is summarized in Algorithm 1. Please note that there are no matrix operations in our algorithm, which means that the algorithm can be implemented at the level of scalar and vector operations. This feature is very important when the algorithm is implemented in chips such as field programmable gate array (FPGA) and digital signal processor (DSP).
**Algorithm 1:** The TDAC algorithm based on NLMS.
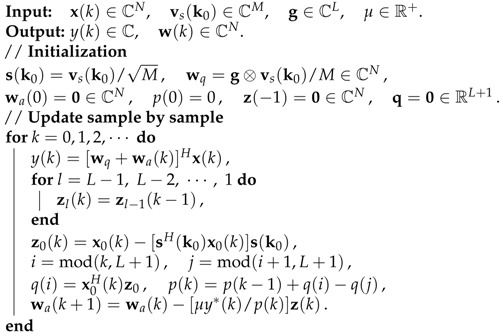


### 3.3. Geometrical Interpretation

**Figure 3:**
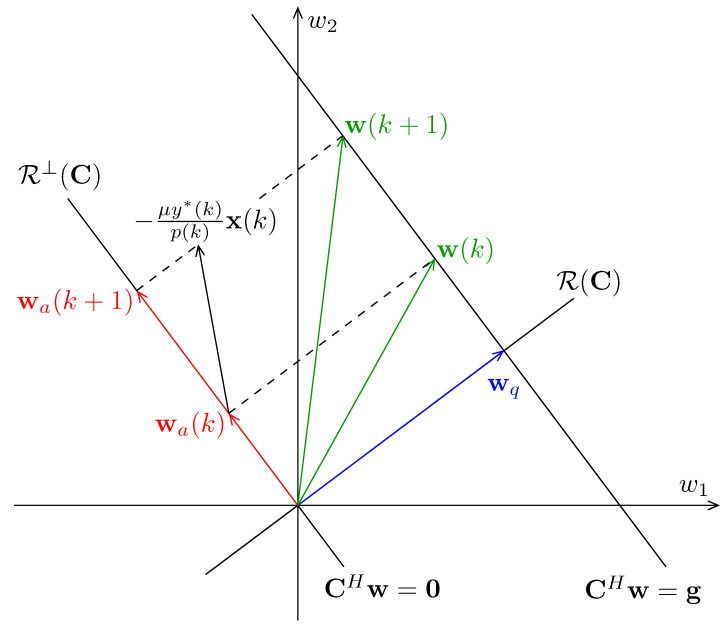
Geometrical interpretation of the proposed algorithm.

The new weight vector update algorithm has a simple geometrical interpretation as shown in Figure 3, where $C^{H}w=0$ and $C^{H}w=g$ define the constraint subspace and the constraint plane, respectively. It follows from (16) that $w_{q}\in R\left(C\right)$. Thus, it is perpendicular to the constraint subspace $R^{⊥}\left(C\right)$. Meanwhile, because $w_{q}$ satisfies the constraint equation $C^{H}w_{q}=g$, it terminates on the constraint plane. In addition, as defined by (20), $w_{a}\left(k\right)$ is the projection of $\tilde{w}\left(k\right)$ onto $R^{⊥}\left(C\right)$. Thus, it lies in the constraint subspace.

From Algorithm 1 we know that only $w_{a}\left(k\right)$ is updated during the iterations. To minimize the output power at the *k*th time instant, an increment $-[\mu y^{*}\left(k\right)/p\left(k\right)]x\left(k\right)$, based on the NLMS criterion, is added to the adaptive vector $w_{a}\left(k\right)$. However, this change may move $w_{a}\left(k\right)$ off the constraint subspace. Thus, the projection of the increment $-[\mu y^{*}\left(k\right)/p\left(k\right)]\mathrm{Px}\left(k\right)$ is used to update $w_{a}\left(k\right)$ to ensure that $w_{a}\left(k\right)$ lies in the constraint subspace.

### 3.4. Computational Complexity Analysis

The computations involved in each iteration of CFA, FDC, FDC-GSC, and TDAC, in terms of complex additions and complex multiplications, are shown in Table 1. We see from the table that our method (TDAC) has the least computations, which is even more efficient than CFA. In contrast, FDC and FDC-GSC increase the weight vector update complexity from O(N) to $O(N^{2})$.

In addition, computing the projection matrix P at the initial stage of FDC requires a complexity of $O(NJ^{2})$, where *J* is the number of constraint points in frequency band. Similarly, computing the blocking matrix B at the initial stage of FDC-GSC requires a complexity of $O(NJ^{2})$. The convolution constraint method has the same update complexity of FDC. However, it needs DFT operations to form the constraint matrix, leading to a higher complexity than FDC. In contrast to FDC and FDC-GSC, the proposed method only requires *N* additions and 2N multiplications at the initial stage.

## 4. Simulation Results

In this section, we present some numerical experiments to compare the performance of the proposed algorithm with other methods. A 10×10 space-time array is employed in the first experiment. More specifically, a uniform linear array (ULA) located along the *z*-axis is considered. The array consists of 10 isotropic antennas whose interelement spacing is 0.5λ where λ is the wavelength corresponding to the carrier frequency. In baseband, each sensor is connected to an FIR filter with 10 TDLs. The distortionless gain vector is set to $g={\left[0,0,0,0,1,0,0,0,0,0\right]}^{T}$. One desired signal and two uncorrelated interferences impinge on the array from $60^{\circ }$, $80^{\circ }$ and $120^{\circ }$, respectively. Noises are assumed to be spatially and temporally uncorrelated zero-mean Gaussian random processes with equal power. The signal-to-noise ratio (SNR) is 10 dB and the interference-to-noise ratios (INR) are 40 dB and 30 dB respectively. Both the signal and interferences occupy a bandwidth of 100 MHz around the carrier frequency of 1000 MHz, i.e., the fractional bandwidth is 10%. We compare our method (TDAC) with the methods of CFA [7], FDC [22], and FDC-GSC [24]. The NLMS-based update Equation (12) with μ=0.02 is used for all of the tested algorithms. For the FDC method, 10 constraint points are uniformly selected in the frequency band.

The performance of broadband beamformer is measured in terms of array output power and signal-to-interference-plus-noise ratio (SINR). The output SINR is defined as

(30) $$\mathrm{SINR}=\frac{w^{H}R_{s}w}{w^{H}R_{i+n}w}\;,$$

where $R_{s}$ is the correlation matrix of the desired signal and $R_{i+n}$ is the correlation matrix of interferences plus noise. We assume that the power spectral densities of the desired signal and the interferences are flat in the bandwidth of considered. We also assume that the signal, interferences, and noise are statistically independent. Under these assumptions, the correlation between the *l*th tap after the *m*th sensor and the *p*th tap after the *q*th sensor for the *d*th impinging wave can be expressed as [35]

(31) $$R_{d}\left(lM+m,pM+q\right)=\sigma_{d}^{2}\exp\left[j\omega_{c}\left(\tau_{q}^{\left(d\right)}-\tau_{m}^{\left(d\right)}\right)\right]\mathrm{sinc}\left[B_{d}\left(\tau_{q}^{\left(d\right)}-\tau_{m}^{\left(d\right)}\right)+B_{d}\left(p-l\right)T_{s}\right]\;,$$

where $\sigma_{d}^{2}$ is the power of the *d*th signal, $\tau_{m}^{\left(d\right)}$ is the propagation delay of the *d*th signal at the *m*th sensor with respect to the origin, and $B_{d}$ is the bandwidth of the *d*th signal. For the simulation scenario described above, $R_{s}=R_{1}$, $R_{i+n}=R_{2}+R_{3}+\sigma_{n}^{2}I_{N}$, where $\sigma_{n}^{2}$ is the noise power.

**Figure 4:**
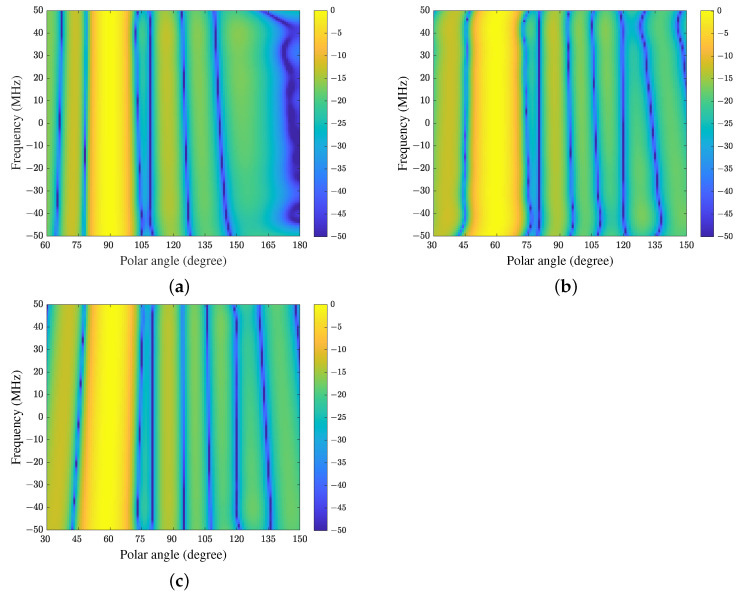
The FWRs of a 10×10 space-time array with a fractional bandwidth of 10%: (**a**) CFA, (**b**) FDC, and (**c**) the proposed method (TDAC).

Figure 4 shows the FWRs of CFA, FDC and TDAC when the adaptive algorithms have converged (for ULA located along the *z*-axis, FWR depends on frequency and polar angle). Because the FWR of FDC-GSC is the same as FDC, it is not shown here. It can be seen that all methods have constant magnitude responses at the constrained direction while placing nulls at the interference directions in the whole frequency band. Because the array is presteered toward the direction of SOI by CFA, the equivalent directions of interferences are also changed. In this example, the equivalent directions of the desired signal and interferences are $90^{\circ }$, $109^{\circ }$ and $180^{\circ }$ respectively, which can be verified in Figure 4a. In addition, we see from Figure 4b,c that the FWR of TDAC is smoother than that of FDC. Hence TDAC may have better SINR performance than FDC in this experiment due to the better sidelobe structure.

**Figure 5:**
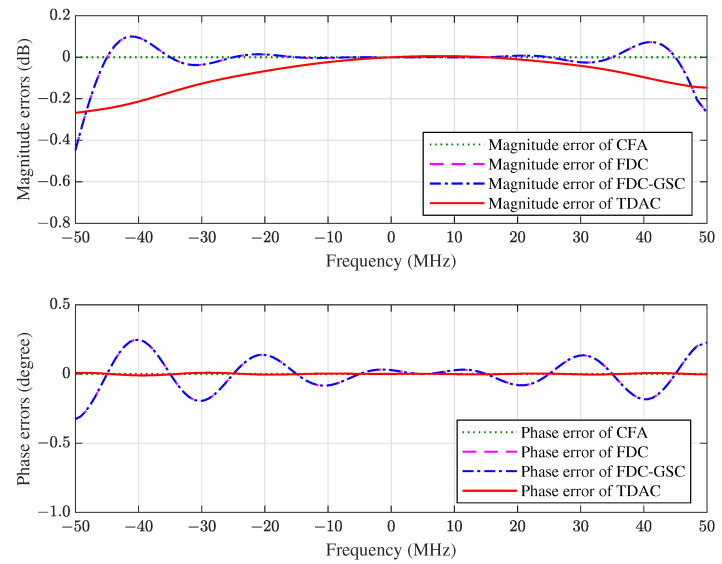
The magnitude and phase errors of the 10×10 space-time array in the direction of SOI.

The magnitude and phase errors in the constrained direction of CFA, FDC, FDC-GSC and TDAC are plotted in Figure 5. It can be seen that (i) the constrained response is equal to the distortionless response when the array is precisely presteered by CFA; (ii) FDC and FDC-GSC have the same frequency response since they are equivalent; (iii) there exist ripples in the frequency response of FDC, this is because only 10 frequency points are constrained by FDC and the responses between these constrained points are not guaranteed; (iv) both FDC and TDAC provide good approximations to the desired frequency response; and (v) TDAC has a smaller phase error than FDC.

**Figure 6:**
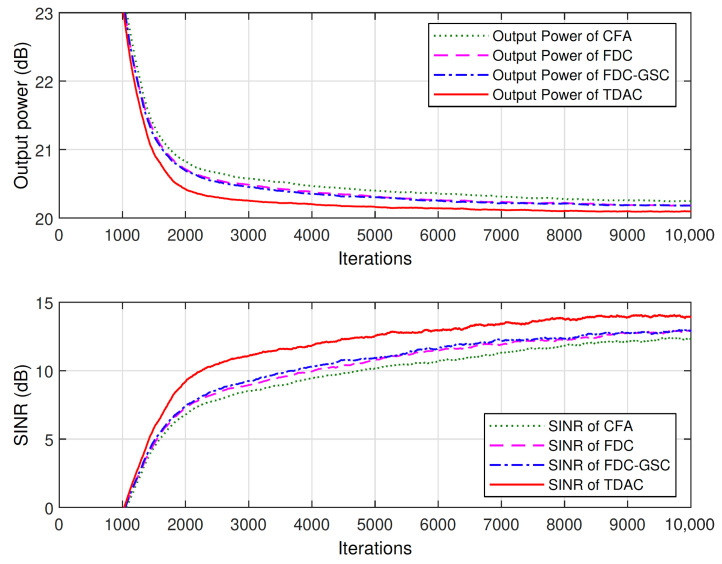
The output power and SINR of the 10×10 space-time array averaged over 100 trials.

Figure 6 shows the learning curves of the output power and SINR of different methods averaged over 100 independent trials. As shown in Figure 6, the four tested methods have the same convergence rate, but our method has the best steady-state SINR performance. The reason CFA has a smaller SINR than other methods is that when the array is presteered toward the direction of SOI, the directions of interferences are also changed. Thus, the array faces a different interference environment, leading to different SINR performance. Although FDC can impose accurate constraints in frequency domain, the frequency responses between the constrained points are not guaranteed, leading to ripples in frequency band as shown in Figure 5. Therefore, similar to the proposed method that approximates the FWR in time domain, FDC is also a type of approximate method that approximates the FWR in frequency domain.

**Figure 7:**
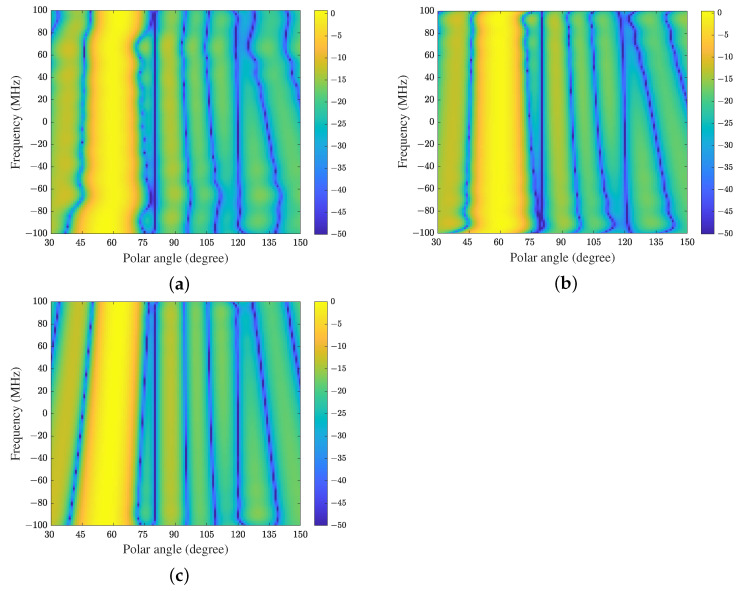
The FWRs of a 10×20 space-time array with a fractional bandwidth of 20%: (**a**) FDC with 20 constraint points (FDC1), (**b**) FDC with 30 constraint points (FDC2), and (**c**) the proposed method.

**Figure 8:**
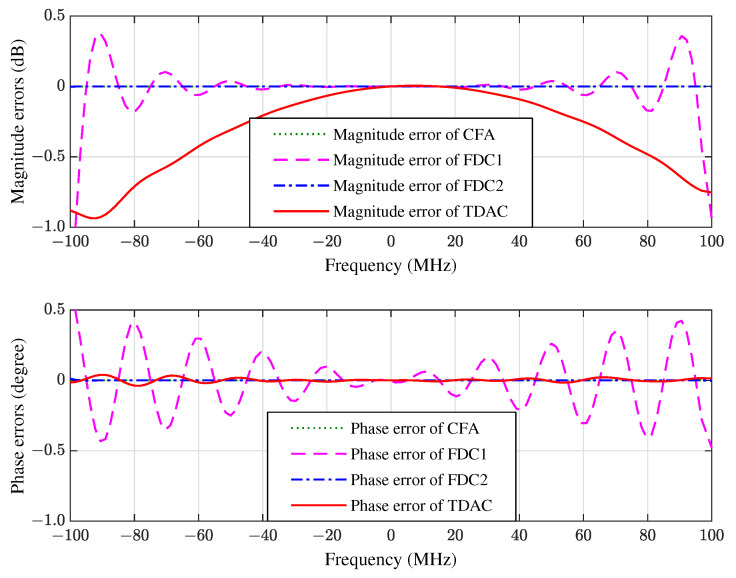
The magnitude and phase errors of the 10×20 space-time array in the direction of SOI.

**Figure 9:**
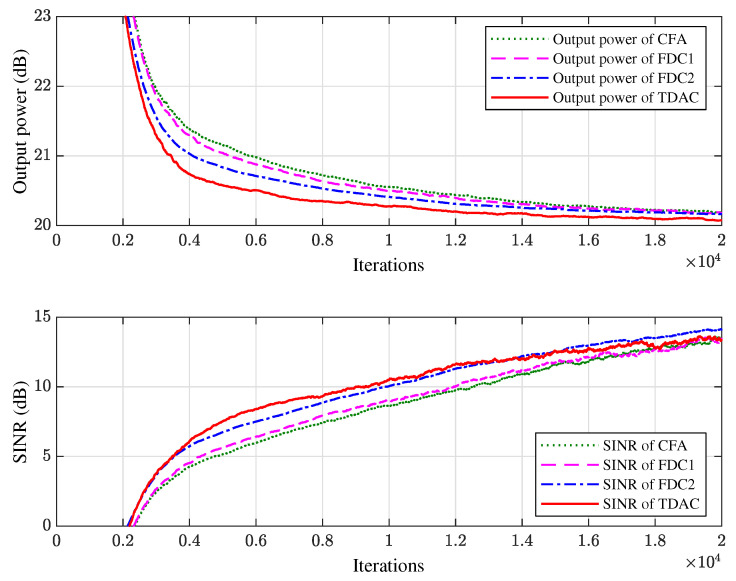
The output power and SINR of the 10×20 space-time array averaged over 100 trials.

In the second experiment we change the bandwidth from 100 MHz to 200 MHz while keeping the carrier frequency fixed at 1000 MHz, i.e., the fractional bandwidth is 20%. We also change the length of FIR filter from 10 to 20, i.e., we use a 10×20 space-time array. The proposed algorithm is compared with the FDC methods with 20 constraint points (denoted by FDC1) and 30 constraint points (denoted by FDC2) respectively. The simulation results of FWRs, constraint errors, and learning curves are shown in Figure 7, Figure 8 and Figure 9, respectively.

## 5. Conclusions

An efficient implementation of the broadband adaptive beamformer without presteering delays was studied in this paper. A new kind of approximate constraint in time domain has been proposed to eliminate the presteering delays of the space-time array. In addition, a new weight vector update algorithm was developed by using the block diagonal structure of the constraint matrix, leading to a computational complexity of O(N) in each iteration. In contrast, the computational complexity of the frequency constraint methods is $O(N^{2})$ in each iteration. Moreover, the new algorithm does not contain matrix operations and can be implemented at the level of scalar and vector operations. This feature is very important for real-time applications, in which the algorithms should be implemented in chips such as FPGA and DSP. Numerical experiments shown that the approximate accuracy of the proposed method is as high as the frequency constraint method for systems with a fractional bandwidth smaller than 10%, while our method has a simpler algorithm structure and a lower computational cost than the existing methods.

## Figures and Tables

**Table 1:** Computations in each iteration of CFA, DFC, DFC-GSC, and TDAC.

Algorithm	Number of Complex Additions	Number of Complex Multiplications
CFA [7]	5N+3M−L−1	4N+3M+1
FDC [22]	$N^{2}+2N+3M-1$	$N^{2}+2N+3M+1$
FDC-GSC [24]	$N^{2}-\left(L-3\right)N-2L-2$	$N^{2}-\left(L-4\right)N-3L+1$
TDAC (proposed)	3N+3M−2	2N+3M+1

## Data Availability

Data sharing not applicable.
